# Haemoglobin concentration on admission to intensive care influences hospital mortality rates and length of stay: a retrospective study

**DOI:** 10.1186/cc12291

**Published:** 2013-03-19

**Authors:** M Adams, P Dean, P Doherty, S Noble, A Mackay

**Affiliations:** 1 Victoria Infirmary, Glasgow, UK

## Introduction

The association between haemoglobin concentrations and mortality has been studied in patients with various comorbidities [1,2]. This study aims to determine the association between haemoglobin levels on admission to intensive care and patient length of stay and mortality.

## Methods

A retrospective collection of data from patient admissions to a single five-bed ICU over a 15-year period identified 5,826 patients between April 1994 and November 2012. Patients were split into groups according to haemoglobin concentration on admission. The data were analysed to determine whether there was any relationship between haemoglobin concentration at ICU admission and any of our outcome measures (unit and hospital mortality, unit and hospital length of stay).

## Results

Patients with haemoglobin concentrations ≤10 g/dl and >10.1 g/dl were used in mortality comparisons. Patients with a haemo globin concentration ≤10 g/dl had an increase in ICU mortality compared with those with haemoglobin levels >10 g/dl (OR = 1.36, 95% CI = 1.30 to 1.71, *P <*0.0001). A similar difference was seen with hospital mortality (≤10 g/dl 37.7% vs. >10 g/dl 27.3%, *P <*0.0001). Unit length of stay was significantly longer in patients with admission Hb ≤10 g/dl (5.34 days) compared with an admission Hb >10 g/dl (4.08 days), *P <*0.0001. Hospital length of stay was also significantly longer in patients with Hb ≤10 g/dl versus Hb >10 g/dl (21.6 days vs. 15.5 days, *P <*0.0001). There was seen to be an inverse correlation between haemoglobin concentration and patient age (*r *= -0.174; *P <*0.0001).

## Conclusion

Haemoglobin concentrations ≤10 g/dl on admission to the ICU are associated with an increase in ICU mortality, hospital mortality, unit length of stay and hospital length of stay when compared with patients admitted with haemoglobin concentrations >10 g/dl.
